# First person – Amy Irving

**DOI:** 10.1242/bio.059477

**Published:** 2022-07-07

## Abstract

First Person is a series of interviews with the first authors of a selection of papers published in Biology Open, helping early-career researchers promote themselves alongside their papers. Amy Irving is first author on ‘ Vitamin D receptor absence does not enhance intestinal tumorigenesis in *Apc^Pirc/+^* rats’, published in BiO. Amy is a research scientist in the lab of Hector DeLuca at the University of Wisconsin-Madison, USA, investigating the individual contributions of sunlight, vitamin D, calcium, and other pathway players on diseases with a location association paradigm, specifically colon cancer and multiple sclerosis.


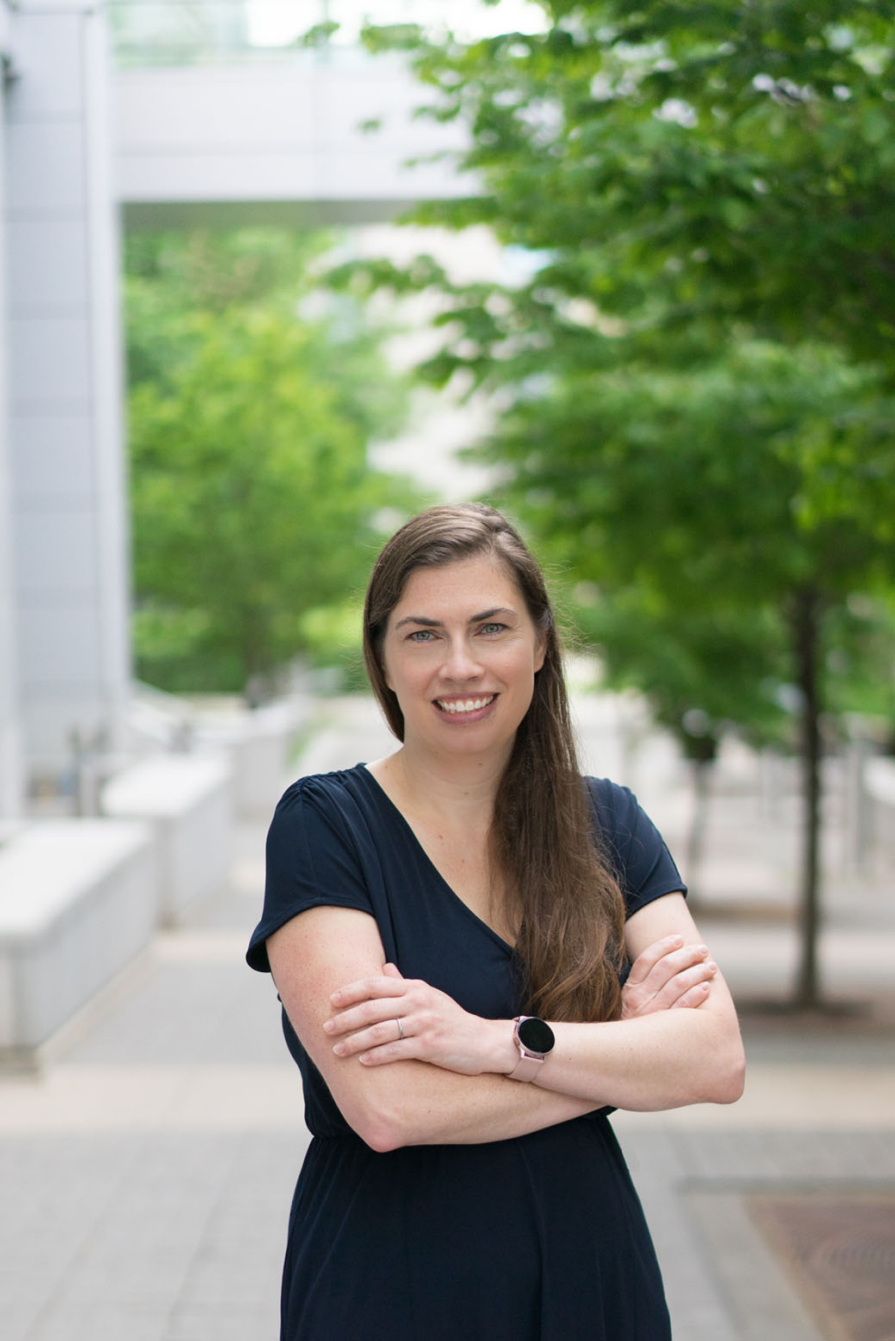


**Amy Irving**

**What is your scientific background and the general focus of your lab?**

My graduate training is in molecular and environmental toxicology and oncology. During these formative years I was lucky enough to also be part of multiple collaborations within biochemistry, which opened the door to my current position. Our lab is deeply invested in unraveling the complex vitamin D network to better understand its contributions to both normal biology and disease development. We are always looking for the right tool to best address the question at hand, using a combination of techniques from molecular biology, chemistry, physiology, and cell biology.

**How would you explain the main findings of your paper to non-scientific family and friends?**

People around the globe have varying susceptibilities to disease, owing to differing genetics, diet, and environmental exposures. Observations made between populations can be an informative place to initiate research questions, but careful dissection of the contributing factors to the observed differences often requires laboratory investigation. Such observations have linked a higher risk for colon cancer with further distance from the equator, and thus lower sunlight exposure. As one of the most well-studied effects of sun exposure, vitamin D has been a prime candidate for this effect. However, vitamin D level in the body is closely linked with circulating calcium – and calcium is very tightly controlled in a narrow concentration window, as it is critical for cellular signaling. This has made the study of any potential role for vitamin D in colon cancer difficult, as other changes often occur when vitamin D is modulated. To combat this, we've approached this question in multiple ways, showing that (1) vitamin D supplementation does not reduce cancer risk in rodent intestinal cancer models (Irving et al., 2011); and (2) vitamin D deficiency does not affect cancer risk when calcium level is controlled (Irving et al., 2018). Our current study further supports these findings, as we have now employed an animal model lacking the receptor for vitamin D and have observed no effect on tumor development, growth, or progression. It is crucial to determine the true molecular players that contribute to colon cancer risk, and to understand how we can modulate our environment in meaningful ways to subsequently reduce that risk.

**What are the potential implications of these results for your field of research?**

We hope that our controlled investigations of vitamin D and colon cancer from multiple angles might encourage those in the field to broaden the search to include other environmental factors that may explain the location association paradigm. Furthermore, diseases other than cancer (e.g. multiple sclerosis) also show a similar epidemiological pattern that thus far have not been convincingly explained by vitamin D. Thus, investigation into other candidates is certainly warranted.

**Figure:**
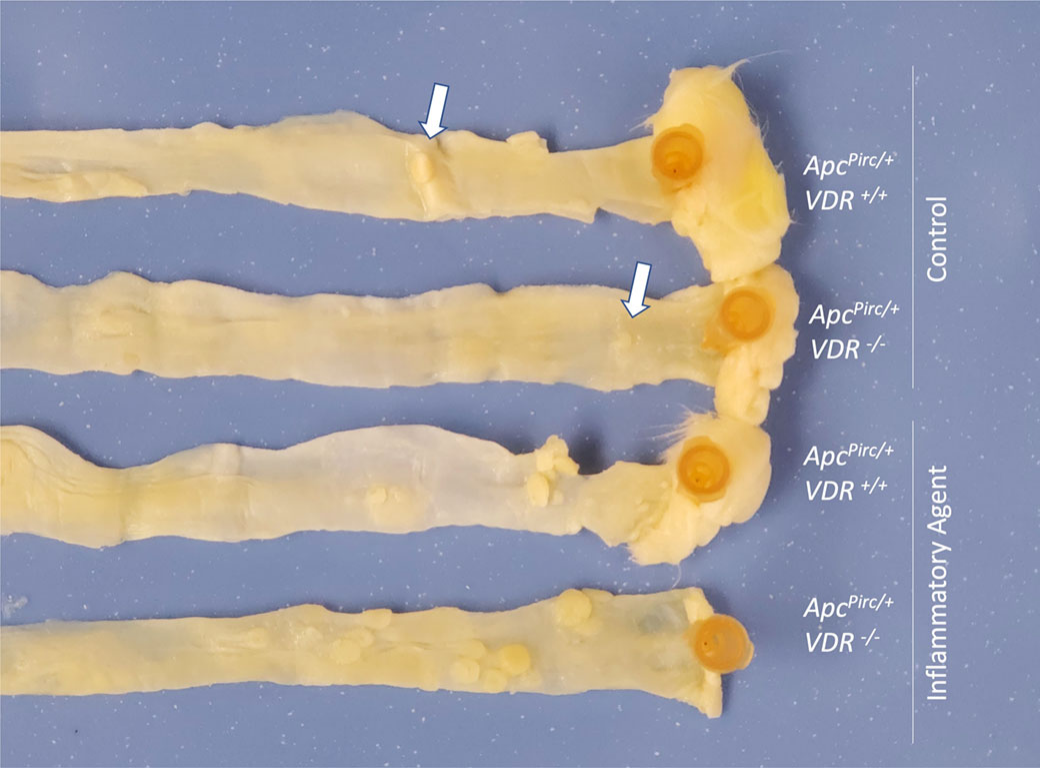
**Distal colonic tumors in female *Apc^Pirc/+^* rats, either expressing or lacking vitamin D receptor (VDR).** Regardless of genotype, control rats develop few tumors (white arrows). When treated with the inflammatory agent dextran sodium sulfate, tumor number increases several-fold; this increase is not affected by the absence of VDR.

**What has surprised you the most while conducting your research?**

In the many ways that we have investigated vitamin D and colon cancer, it has surprised me that we have only seen an effect on tumorigenesis when calcium is also perturbed. I think there is definitely more to this story!

**What, in your opinion, are some of the greatest achievements in your field and how has this influenced your research?**

Modern genome editing technologies, including CRISPR/Cas, have opened new avenues for modeling in the rat. Historically, the rat was the favored model species for studies of physiology and toxicology, owing to similarities with humans. Despite this, the mouse offered advantages in genome manipulation far ahead of the rat, boosting its use in the laboratory. While the *Apc^Pirc/+^* rat model demonstrates features of the human disease which are lacking in similar *Apc* mutant mouse models, until recently it was a cumbersome process to layer additional genetic alterations in the rat. The flexibility and speed at which new genetic models can now be developed in the rat will allow us to investigate questions in multiple genera and, in turn, tell us more about the human disease.

“The flexibility and speed at which new genetic models can now be developed in the rat will allow us to investigate questions in multiple genera and, in turn, tell us more about the human disease.”

**What changes do you think could improve the professional lives of early-career scientists?**

I think that graduate programs could better prepare future scientists for the inherent challenge of doing good science. We are highly trained in laboratory techniques, critical thinking, and the scientific process, culminating in solid publications. But every good scientist will encounter hurdles on their journey to discover, and this is normal and expected. Mentally this can be the most challenging part of a scientific career, and rather than viewing tribulations as setbacks, they should be accepted as progress. Hopefully, the gradual change we are seeing as more journals accept publications with negative findings will shift our view on what is successful science. Carving a new path forward isn't easy. In the wise words of my mentor Hector DeLuca, “If it were easy, we'd already understand the questions we are working so diligently to answer”.

**What's next for you?**

My research is dedicated to uncovering the molecular basis for the observed protection offered by sunlight on diseases such as colon cancer and multiple sclerosis, which are both highly prevalent and often affect those in their prime. The public health implications for such a discovery could be significant.
